# Melanoacanthoma: a potential pitfall of reflectance confocal microscopy^☆^^☆☆^

**DOI:** 10.1016/j.abd.2019.01.002

**Published:** 2019-10-26

**Authors:** Ana Carolina Souza Porto, Tatiana Pinto Blumetti, Mariana Petaccia de Macedo, Juliana Casagrande Tavoloni Braga

**Affiliations:** Cutaneous Oncology Department, AC Camargo Cancer Center, São Paulo, SP, Brazil

**Keywords:** Confocal microscopy, Dermoscopy, Diagnosis, Melanoma, Seborrheic keratosis

## Abstract

Melanoacanthoma is a rare variant of seborrheic keratosis, which is notable for dark pigmentation and fast radial growth, making it difficult to distinguish from melanoma. Histologically, it is characterized by proliferation of keratinocytes and dendritic melanocytes. The authors report a scalp lesion, fast growing, suspected by dermoscopy and confocal microscopy examination, with dendritic cells distributed throughout the lesion. Based on these findings, it was not possible to classify this lesion as clearly benign, so it was excised. Histopathologic evaluation and immunostain were consistent with melanoacanthoma.

## Introduction

Cutaneous melanoacanthoma (CM) is a rare variant of seborrheic keratosis (SK) that occurs mainly in fair-skinned adults, preferentially in the head, neck, and chest.^1^, ^2^ This benign lesion is clinically notable for its dark pigmentation and fast-radial growth, making this entity difficult to distinguish from melanoma. CM has been seldom characterized with dermoscopy and with reflectance confocal microscopy (RCM).^3^

## Case report

**Figure 1:**
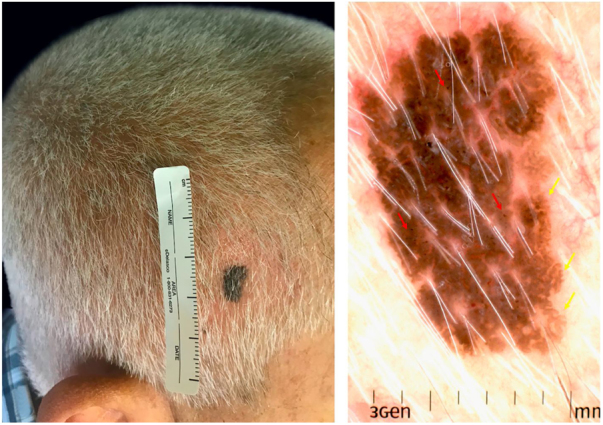
Clinical and dermatoscopic image of melanoacanthoma. (A) Clinical view: 9-mm asymmetric, dark-brown, sharply demarcated plaque on the right parietal. (B) Dermoscopic image with polarized light: presence of irregularly distribute dots (red arrow) and fat fingers (yellow arrow).

A 76-year-old male patient presented a rapidly growing 9-mm asymmetric, dark-brown patch on the scalp. Dermoscopic examination showed comedo-like openings, fat fingers, and irregularly distributed dots (Fig. 1).

**Figure 2:**
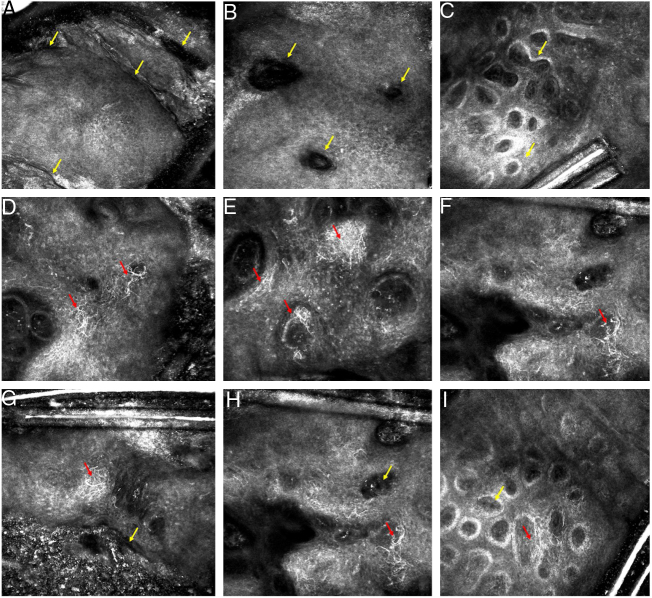
(A–I) Reflectance confocal microscopy of melanoacanthoma. (A–C) Findings suggestive of seborrheic keratosis (SK). (A) At the suprabasal epidermis level, a regular honeycomb pattern and epidermal projections (yellow arrow). (B) Keratin filled invaginations (yellow arrow). (C) At the dermo-epidermal junction (DEJ) level, densely packed round-to-polymorphous dermal edges (yellow arrow). (D–E) Findings suspected for melanoma. (D) At the suprabasal epidermis, a regular honeycomb pattern and dendritic pagetoid cells (red arrow). (E and F) At the DEJ level, an infiltration of dendritic cells around and between the dermal papillae (red arrow). (G–I) Findings present in SK (yellow arrow) and melanoma (red arrow).

The first diagnostic hypothesis was SK, but the irregularly distributed dots lead the authors to perform RCM to exclude melanoma. It showed a typical honeycomb pattern at the spinous-granular layer, epidermal projections, keratin filled invaginations, and widespread dendritic pagetoid cells. At the dermo-epidermal junction (DEJ), densely packed polymorphous dermal edged papillae were observed, in addition to an infiltration of dendritic cells between dermal papillae. At the papillary dermis level, melanophages were observed (Fig. 2). Based on RCM findings, it was not possible to classify this lesion as clearly benign, so it was excised.

**Figure 3:**
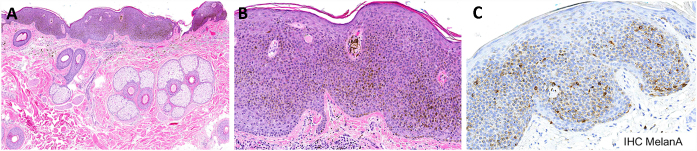
Histopathologic analysis and immunohistochemical stainings of the melanoacanthoma. (A) A hyperplastic epidermis, hyperkeratosis, and intense acanthosis. (B) Intermingled with the keratinocytes, dendritic melanocytes were present throughout the lesion. (C) Immunohistochemistry study confirming an increased density of melanocytes cells. (A and B) Hematoxylin & eosin stain; (C) Melan-A staining; original magnifications: A, x40, B–C, x400.

On histopathological evaluation, hyperplastic epidermis, hyperkeratosis, acanthosis, and dendritic melanocytes were seen throughout the lesion. Melan-A and CD1a immunostain were positive and confirmed an increased density of melanocytes and Langerhans cells (Fig. 3). These findings were consistent with CM.

## Discussion

CM is an uncommon neoplasm histologically characterized by proliferation of epidermal keratinocytes and large dendritic melanocytes. The melanocytes are scattered throughout the tumor.^3^, ^4^, ^5^ The exact incidence of CM among the SK histopathological subtypes is unclear. Roh et al. found a high percentage (9.2%) in their study and refuted that CM is extremely rare.^6^

Four case reports have previously described dermoscopic appearance of CM, and features of both benign as well as malignant lesions have been reported. Rossiello et al. described a starburst pattern,^7^ while Shankar et al. observed a pattern of ridges and fissures.^4^ Chung et al. described features characteristic of SK in eight CM cases; comedo-like openings and sharp demarcation were the most common findings. However, melanoma-specific dermoscopic criteria were identified in six of these cases, and blue-white veil and atypical dots were the most frequent features.^1^ In the most recent report, Shahriari et al. described a black papule with blue-black pigmentation, suspicious for nodular melanoma.^3^

The assessment of the reported lesion with polarized light (PL) dermatoscopy demonstrated comedo-like openings, fat fingers, and irregularly distributed dots. PL offers a better view of structures located deeper into the skin, whereas non-polarized light allows a better visualization of more superficial structures. With PL, milia-like cysts are less visible; the melanin appears sharper and darker, and comedo-like openings can be interpreted as dots. Thus, the use of PL instruments, increasingly popular among dermatologists, may hinder the diagnosis of CM.

In equivocal clinical and dermoscopic SK cases, Ahlgrimm-Siess et al. showed that at least three RCM criteria for SK diagnosis can be found in almost of all lesions; including epidermal projections, keratin-filled invaginations, and corneal pseudocysts at the suprabasal levels; densely packed polymorphous dermal papillae at the DEJ level. Furthermore, there is absence of any features suggestive of malignancy.^8^

On RCM examination, the presence of pagetoid spread of dendritic melanocytes within the epidermis, as well the presence of these cells at DEJ, is an important clue for melanoma, in particular the lentigo malign subtype.^9^ The presence of numerous bright dendritic cells in the supra-basal epidermis can also be found in pigmented squamous cell carcinoma *in situ*.^10^ Summing up, if dendritic cells are seen scattered throughout a lesion on RCM, it is expected that the dermatologist will be suspicious and quickly jump to conclusion of malignancy.

RCM features of CM have been described in only one case by Shahriari et al. The authors observed numerous, tangled dendritic cells, at the basal and supra-basal layers. This pattern was interpreted as suspicious for melanoma and the lesion was excised, confirming CM.^3^

In the present case, a regular honeycomb and polymorphous papillae were identified, as well as an infiltration of dendritic cells at the basal and supra-basal layers and at the DEJ level. Concerned with these diffuse dendritic cells, even in the presence of other RCM criteria of benignity, the authors performed a biopsy. In hindsight, dendritic cells scattered throughout the tumor in the presence of RCM criteria for SK are consistent with the histopathological description of CM.

In conclusion, ruling out melanoma was difficult after RCM imaging in the present case, as it was for Shahriari et al. The present authors believe that the appearance of uniform dendritic melanocytes throughout the lesion, in the context of a thickened epidermis with RCM features of SK, may be a possible clue of CM. Reports of more cases analyzed with RCM and the definition of criteria for diagnosis of this benign tumor is necessary in order to prevent CM from being a pitfall of RCM examination.

## Financial support

None declared.

## Author's contributions

Ana Carolina Souza Porto: Approval of the final version of the manuscript; conception and planning of the study; elaboration and writing of the manuscript; obtaining, analyzing and interpreting the data; effective participation in research orientation; intellectual participation in propaedeutic and/or therapeutic conduct of the cases studied; critical review of the literature; critical review of the manuscript.

Tatiana Pinto Blumetti: Effective participation in research orientation; intellectual participation in propaedeutic and/or therapeutic conduct of the cases studied; critical review of the literature; critical review of the manuscript.

Mariana Petaccia de Macedo: Effective participation in research orientation; intellectual participation in propaedeutic and/or therapeutic conduct of the cases studied; critical review of the literature; critical review of the manuscript.

Juliana Casagrande Tavoloni Braga: Effective participation in research orientation; intellectual participation in propaedeutic and/or therapeutic conduct of the cases studied; critical review of the literature; critical review of the manuscript.

## Conflicts of interest

None declared.
